# Isoenergetic reduction of dietary macronutrients modulates respiratory quotients and heat increment of feeding but not energy expenditure in cats

**DOI:** 10.1093/jas/skaf081

**Published:** 2025-03-12

**Authors:** Hannah Godfrey, Anna Kate Shoveller, Janelle Kelly, Darcia Kostiuk, Jennifer Saunders Blades, Adronie Verbrugghe

**Affiliations:** Department of Biomedical Sciences, Ontario Veterinary College, University of Guelph, Guelph, Ontario, Canada N1G 2W1; Department of Animal Biosciences, Ontario Agricultural College, University of Guelph, Guelph, Ontario, Canada N1G 2W1; Champion Petfoods Holdings, Morinville, Alberta, Canada T8R 1K7; Champion Petfoods Holdings, Morinville, Alberta, Canada T8R 1K7; Champion Petfoods Holdings, Morinville, Alberta, Canada T8R 1K7; Department of Clinical Studies, Ontario Veterinary College, University of Guelph, Guelph, Ontario, Canada N1G 2W1

**Keywords:** carbohydrates, energy metabolism, fat, feline nutrition, indirect calorimetry, protein, thermic effect of feeding

## Abstract

Indirect calorimetry can provide insights into the metabolic processes occurring in cats through substrate utilization and energy expenditure (**EE**). Additionally, the influence of dietary macronutrients on the heat increment of feeding (**HIF**) in cats remains unexplored. As such, this proof of principle study aimed to test the short-term effects of 3 test diets formulated for adult maintenance according to AAFCO by comparatively reducing protein, fat, or carbohydrates, to create a low-protein (**LP**; protein 28%ME, fat 40%ME, nitrogen-free extract (**NFE**) 28%ME), low-fat (**LF**; protein 40%ME, fat 27%ME, NFE 27%ME), and a low-carbohydrate (**LC**; protein 35%ME, fat 40%ME, NFE 20%ME) diet on respiratory quotients (**RQ**), EE, and HIF in cats. Adult (3.4 ± 0.1 yr of age), male, neutered cats (*n* = 12) were assigned to 1 of 3 groups and offered each diet at an amount to maintain body weight (**BW**) for 2 wk in a 3 × 3 Latin square design. The cats BW ranged from 3.94 to 6.33 kg (mean ± SEM BW of 5.08 ± 0.28 kg) and body condition score (**BCS**) ranged from 4/9 to 7/9 (mean ± SEM BCS of 5.58 ± 0.38). At the end of each test period, 22-h indirect calorimetry was performed to determine RQ, EE, and HIF. Daily food intake was greater for cats consuming the LF diet (61.6 ± 1.0 g/d) compared to the LP (58.3 ± 1.0 g/d) and LC (57.7 ± 1.0 g/d) diets (*P* < 0.0001), though energy intake was similar between diets (223.8 ± 2.2, 227.9 ± 2.0, and 228.4 ± 2.0 kcal/d) (*P* = 0.1191). In the fasted, immediate postprandial (0 to 120 min), and postabsorptive (0 to 1,200 min) states, cats fed LC had a lower RQ compared to LF (*P* = 0.0154, *P* = 0.0346, and *P* = 0.0407, respectively), indicating greater fat oxidation when consuming LC. No differences were observed between the LP diet and the LF and LC diets for RQ (*P* > 0.05). For all cats regardless of diet, the RQ increased from the immediate postprandial to postabsorptive state (*P* < 0.0001) as expected. Following feeding, EE increased for all cats regardless of diet (*P* < 0.0001). No diet effect was observed for EE in the fasted, immediate postprandial, or postabsorptive states. Diet did not affect HIF in the immediate postprandial state; however, the LP diet had a lower HIF compared to the LF diet over the entire post-feeding state when using the National Research Council ME equation, though the HIF was similar between the LC diet to the LP and LF diets (*P* = 0.0360). Future research should explore the long-term effects of low levels of dietary macronutrients in cat foods and their role in energy metabolism under various energy balance conditions.

## Teaser

This study investigates the effects of low dietary macronutrients on respiratory quotients, energy expenditure, and the heat increment of feeding in domestic cats to deepen the understanding of macronutrient influences on metabolism. Macronutrient distribution did not affect energy expenditure, though a low-carbohydrate diet enhanced fat oxidation and the heat increment of feeding was lower in cats consuming a low-protein diet.

## Graphical Abstract

**Graphical Abstract:**
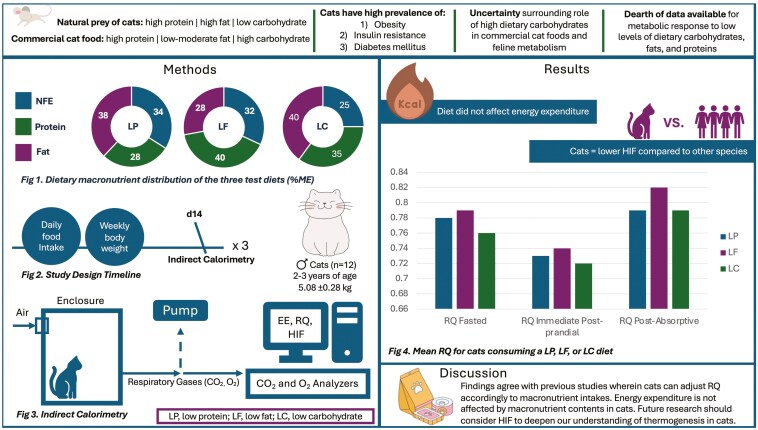

## Introduction

The continuously high prevalence of feline obesity is an ongoing health and welfare concern for domestic cats. Age, sex, and breed may affect obesity risks in cats; however, indoor confinement, physical activity, gonadectomy, and feeding management also appear to have a role (Scarlett and Donoghue, 1998; Lund et al., 2005; German, 2006; Colliard et al., 2009; Courcier et al., 2010; Serisier et al., 2013; Rowe et al., 2015, 2017). In recent years, the consumption of extruded dry foods, which are often high in digestible carbohydrates (20% to 40 % metabolizable energy; **ME**), has been suggested as a risk factor for feline obesity (Buffington, 2008; Villaverde and Fascetti, 2014; Rowe et al., 2015). The concern surrounding the high digestible carbohydrate content in dry foods is largely driven by the nutritional idiosyncrasies of the domestic cat, an obligate carnivore, compared to omnivorous species such as humans or dogs. Regardless of the metabolic differences in the carnivorous cat, cats do have an ability to adjust the rate of carbohydrate oxidation and gluconeogenesis for the maintenance of blood glucose concentrations (Hoenig et al., 2007; Green et al., 2008; Gooding et al., 2014). In addition, the consumption of energy above energy requirements, often driven by dietary fat, appears to be a greater risk factor for obesity in cats (Nguyen et al., 2004a; Backus et al., 2007).

Presently, studies have solely focused on the effects of high levels of dietary protein, fat, or carbohydrates, referring to nitrogen-free extract (**NFE**), when fed ad libitum (Nguyen et al., 2004b; Backus et al., 2007; Wei et al., 2011; Coradini et al., 2014; Gooding et al., 2014, 2015). Few of these studies have assessed energy expenditure (**EE**) and substrate utilization (Nguyen et al., 2004a; Wei et al., 2011; Gooding et al., 2014). A low EE can be a risk for excess weight gain, specifically when coupled with free-feeding practices. Consumption of a food high in dietary fat resulted in a lower EE compared to high-carbohydrate intakes, whereas a high-protein food was observed to increase EE compared to high-carbohydrates, though it is unclear whether this was due to greater energy intake or due to the differences in macronutrient content (Wei et al., 2011; Gooding et al., 2014). Further, substrate utilization determined via respiratory quotient (**RQ**) can offer insight into the metabolic alterations in response to changes in dietary macronutrient concentrations. An increase in RQ suggests less fatty acid oxidation and greater carbohydrate oxidation.

To the authors’ knowledge, current studies have utilized a 2-diet approach, comparing high-carbohydrate to high-fat foods (Gooding et al., 2014), or to high-protein foods (Nguyen et al., 2004b; Wei et al., 2011). Verbrugghe et al. previously used a 3-diet approach wherein a pairwise isoenergetic reduction of each macronutrient was incorporated to test the individual effect of each energy source on the glucose and insulin postprandial response in cats (Verbrugghe et al., 2010). However, this approach has not previously been used to investigate the separate role of each macronutrient on EE and substrate utilization in cats when fed for maintenance. Further, the EE can be used to calculate the heat increment of feeding (**HIF**), a variable that, to the authors’ knowledge, has only been investigated in one feline study (Asaro et al., 2019). Therefore, the HIF for cats and its response to dietary macronutrients requires further investigation. This proof of principle study aimed to deepen the understanding of how lowered levels of macronutrients in the diets of cats, formulated using a pairwise, isoenergetic reduction of macronutrient approach (Verbrugghe et al., 2012), affects RQ, EE, and the HIF when fed to maintain body weight (**BW**). Additionally, the present study will utilize both the industry standard, modified Atwater (AAFCO, 2021), and the National Research Council (**NRC**) methods to calculate the ME to compare differences between these methodologies on the study findings, which has not previously been investigated in terms of HIF (%ME). It is hypothesized that RQ values support previous findings such that cats can adjust substrate utilization in response to dietary macronutrient intakes. Further, macronutrient distribution in the diet for cats does not directly affect EE and, as such, changes in EE are not expected when fed to maintain BW during an acute exposure. Cats, as obligate carnivores, will have HIF values representative of other carnivorous species (Smith et al., 1978; Asaro et al., 2019), and will respond to dietary macronutrient intakes following the hypothesis that dietary protein elicits an increase in HIF compared to dietary fats (Smith et al., 1978; Westerterp, 2004).

## Materials and Methods

All experimental procedures were approved by the University of Guelph Animal Care Committee (AUP #4865) and were in accordance with national and institutional guidelines for the care and use of animals in research.

### Animals and housing

Twelve male neutered domestic shorthair colony cats aged 2 to 3 yr (mean age of 3.4 ± 0.1 yr) from Marshall’s Bio Resources (Waverly, NY, USA) were enrolled and group-housed in a free-living environment (7 m × 5.8 m) at the Animal Biosciences Cat Colony at the University of Guelph (Guelph, ON, Canada). Prior to enrollment, the cats BW ranged from 3.94 to 6.33 kg (mean ± SEM BW of 5.08 ± 0.28 kg), and body condition score (**BCS**) ranged from 4/9 to 7/9 (mean ± SEM BCS of 5.58 ± 0.38) (Laflamme, 1997). All cats were deemed healthy based on a veterinary physical exam, complete blood count, and serum biochemical profiles.

The cats had access to various enrichment materials: scratching posts; cat trees; hiding boxes; beds; toys; and perches; with ad libitum access to water. Daily human interactions such as voluntary brushing, playing, and petting were provided at a maximum of 2-h/d from 1300 to 1500 hours. The room was maintained at a mean temperature of 24.3 ± 0.05 °C and mean humidity 40.3 ± 0.9% with a 12-h light cycle (0700 to 1900 hours). Daily cleaning and sanitization were conducted, and litter boxes were emptied twice per day.

### Dietary treatments

Three extruded test diets were formulated for cat adult maintenance in accordance with the Association of American Feed Control Officials (**AAFCO**) nutrient profile (Table 1) (AAFCO, 2023). Following a similar approach to Verbrugghe et al. (2012), the same ingredients were used and adjusted to create a pairwise change in macronutrient content to create 3 diets with relatively reduced macronutrients to each other, thereby forming a low-protein (**LP**), a low-fat (**LF**), and low-carbohydrate (**LC**) diet with similar vitamin and mineral concentrations, total dietary fiber, and physical structure. The diets were formulated such that the LC diet (target: protein 40% ME; fat 42% ME; NFE 18 %ME) differed from the LF (target: protein 42 %ME; fat 28 %ME; NFE 30%) and the LP (target: protein 28 %ME; fat 42%; NFE 30 %ME) diets by isoenergetic substitution (%ME) of dietary carbohydrate, referring to NFE, for dietary fat and dietary protein for dietary carbohydrates, respectively, using the modified Atwater coefficients (Verbrugghe et al., 2012). Therefore, the LC diet is low in carbohydrates relative to the LF and LP; the LF diet is low in fats relative to the LP and LC; and the LP diet is low in protein relative to the LF and LC.

**Table 1.** Ingredient list and analyzed nutrient content of an LP, LF, and LC extruded test diets formulated for adult maintenance in an isoenergetic approach^1^

	LP	LF	LC
*Ingredients, %*			
Chicken meal	17.6	18	18.4
Fresh chicken	20.0	5.0	8.0
Dried chicken	1.1	17.1	14.5
Pea starch	19.8	17.8	6.8
Pea fiber	10.9	12.0	11.9
Egg powder	6.0	2.0	8.0
Poultry hydrolysate	0.5	7.7	8.0
Oat groats	7.5	10.6	9.7
Chicken fat	6.8	2.2	6.0
Fish oil	2.1	0.7	1.9
Dry palatant	1.3	1.3	1.3
Liquid palatant	3.0	3.0	3.0
Vitamin/mineral premix	2.7	1.7	1.7
Kelp	0.3	0.5	0.4
Salt	0.4	0.4	0.4
*Nutrient profile, % as fed*			
Moisture	8.6	5.6	6.3
Dry matter	91.4	94.5	93.7
Crude protein	31.3	42.0	39.9
Crude fat	18.2	12.4	18.5
Crude fiber	4.6	5.5	5.3
Total dietary fiber	11.2	11.8	12.4
Ash	6.2	7.4	7.0
NFE^2^	39.7	32.7	29.3
ME_Atwater_ (kcal/kg)	4,032.0	3,668.5	3,994.5
ME_NRC_ (kcal/kg)	4,609.5	3,919.7	4,197.0
*Macronutrient distribution, % ME*_*Atwater*_ ^3^			
Protein	28.0	40.0	35.0
Fat	38.0	28.0	39.5
NFE	34.0	32.0	25.5
*Macronutrient Distribution, % ME*_*NRC*_ ^4^			
Protein	23.8	37.5	33.3
Fat	33.6	26.9	37.5
NFE	30.1	29.2	24.4

^1^LC = low-carbohydrate, LF = low-fat, LP = low-protein, ME = metabolizable energy, NFE = nitrogen-free extract.

^2^Calculated as: Nitrogen-free extract = 100 − (crude protein + crude fat + crude fiber + ash) (Equation 1) (National Research Council, 2006).

^3^Calculated ME using modified Atwater Equation (Equation 2) (AAFCO, 2021).

^4^Calculated ME using NRC Predictive Equations (Equation 3) (National Research Council, 2006).

A proximate analysis (Bureau Veritas, Mississauga, ON, Canada) was conducted on each of the 3 test diets using the appropriate methods as outlined by the Association of Official Analytical Chemists (**AOAC**) and the American Oil Chemist Society (**AOCS**) for moisture (AOAC 935.29), crude protein (AOAC 992.15), crude fat (AOAC 922.06, AOAC 933.05), crude fiber (AOCS Ba 6a-0.5), ash (AOAC 923.03), and total dietary fiber (AOAC 991.43, 985.29) (Cunniff and Association of Official Analytical Chemists, 1995). The NFE and ME (ME_Atwater_) were calculated using the following equations (National Research Council, 2006; AAFCO, 2021):


$$\begin{array}{lll} NFE\;(\%)= & 100-[Crude\;protein\;(\%)-Crude\;fat\;(\%)-Crude\;f\;\;ibre\;(\%)\; & -Ash\;(\%)-Moisture\;(\%)] \end{array}$$

$$\begin{array}{l} ME=10*\left[(Crude\;\;\;protein*3.5)+(Crude\;\;\;fat*8.5)*(NFE*3.5)\right] \end{array}$$

Additionally, proceeding the study, the ME was calculated using the NRC calculation (ME_NRC_) as follows (National Research Council, 2006):


$$\begin{array}{lll} Step\;1: & Gross\;Energy\;\left(GE,\;kcal\right)=\left(5.7\times g\;protein\right)+\left(9.4\times g\;fat\right)\; & +4.1\times \left(g\;NFE+g\;crude\;f\;\;ibre\right) \end{array}$$

$$\begin{array}{l} Step\;2:\;Energy\;Digestibility\;\left(\%\right)=87.9-\left(0.88\times \%\;crude\;f\;\;ibre\right) \end{array}$$

$$\begin{array}{lll} & Step\;3:Digestible\;Energy\;\left(DE,\;\frac{kcal}{g}\right)\; & =\left(GE\times \frac{Energy\;digestibility\left(\%\;dry\;matter\right)}{100}\right) \end{array}$$

$$\begin{array}{l} Step\;4:ME\left(\frac{kcal}{g}\right)=DE-\left(0.77\times g\;protein\right) \end{array}$$

### Experimental design

The cats were assigned to 1 of 3 groups, balanced for BW and BCS, for a 3 × 3 Latin square design such that each group received each test diet (LP, LF, or LC) for 2 wk (14 d) in random order. Cats were offered their allotted food for 1 h/d in individual cages from 0800 h to 0900 h. The food offered and food remaining were weighed and recorded to document daily food intake and each cat’s BW was measured weekly. Using both calculated ME (ME_Atwater_ and ME_NRC_) (Table 1), cats were provided the respective test diet to meet their individual maintenance energy requirements according to historical colony energy intake data. On day 14 of each test period, 22-h indirect calorimetry was performed to determine RQ, EE, and HIF.

### Indirect calorimetry

Indirect calorimetry analyses to assess RQ, EE, and HIF in response to LP, LF, or LC diets were conducted on day 14 of each test period. Cats were previously acclimated to indirect calorimetry sessions using published protocols (Gooding et al., 2012). Each indirect calorimetry session was 22-h in length comprising of a 30 min period of gas equilibrium, a 1.5-h fasted period, an immediate postprandial period (2-h), and 18-h postabsorptive state. Following the fasted period, cats were provided 30 min to consume their allotted food, after which the immediate postprandial period commenced.

Indirect calorimetry and calibration of gases were conducted as previously described (Godfrey et al., 2022; Rankovic et al., 2022, 2023). Briefly, an open circuit, ventilated system wherein air was pulled into and through the plexiglass chamber measuring 53 cm × 53 cm × 79 cm (length × width × height) at set flow rates for each individual cat (2.0 to 6.5 L/min; Qubit C950 Multi Channel Gas Exchange, Qubit Systems Inc., Kingston, ON, Canada) was used. To calculate RQ and EE, the following calculations were used (Jequier et al., 1987).


$$\begin{array}{l} T1.\;RQ=\frac{CO_{2}\;\;\;produced\;\left(L\right)}{O_{2\;\;\;}consumed\;\left(L\right)} \end{array}$$

$$\begin{array}{lll} & 2.\;EE\;(kcal)=3.94*O_{2}\;consumed\;(L)\; & +1.11*CO_{2}\;produced\;(L) \end{array}$$

Last, following previous methods in cats (Asaro et al., 2019) the resting fed metabolic rate (**RFMR**) was characterized as the lowest observed value of EE (kcal/d) by each cat (Blaxter, 1989).

### Heat increment of feeding

The HIF was calculated for the immediate postprandial state (0 to 120 min) and for the entire post-feeding state (0 to 1,200 min) by calculating the difference in area under curve (**AUC**) for postabsorptive total EE and the RFMR (National Research Council, 2006; Asaro et al., 2019). Using the food and energy intake at the time of calorimetry, HIF was calculated as % of ME_Atwater_ and as % of ME_NRC_, and as kcal per 100 g on a dry matter (**DM**) basis for both time ranges.

### Statistical analyses

All data were statistically analyzed via SAS Studio 3.8 (SAS Institute, Cary, NC, USA). The AUC for RQ values and for the HIF calculations were determined using Prism (GraphPad Software, Boston, MA, USA) via the trapezoidal method for fasted (−60 to 0 min), immediate postprandial (0 to 120 min), and the postabsorptive periods (120 to 360, 360 to 720, and 720 to 1,200 min) states for RQ and for the immediate postprandial (0 to 120 min) and postabsorptive period (0 to 1,200 min) for HIF, respectively. Both RQ and EE were analyzed for the fasted (−60 to 0 min), immediate postprandial (0 to 120 min), and postabsorptive periods (0 to 1,200 min) states. Additionally, mean RQ for each treatment was pooled and further assessed for the fasted (−60 to 0 min), immediate postprandial (0 to 120 min), and the postabsorptive periods (120 to 360, 360 to 720, and 720 to 1,200 min) states. Data were checked for normality of the residuals using the Shapiro–Wilk test and log transformation was used as necessary to meet the assumptions of ANOVA. The proc GLIMMIX procedure, with test diet (LP, LF, or LC) considered as a fixed effect, period as a random effect, and cat as subject was used. Time was added as a repeated measure when appropriate. A Tukey post hoc adjustment using the covariance structure that resulted in the smallest Akaike information criterion value was used to separate means when the fixed effect was significant. Data for EE was pooled for all cats from all diets and differences in EE over time were compared for fasted (−60 to 0 min), immediate postprandial (0 to 120 min), and the postabsorptive periods (120 to 360, 360 to 720, and 720 to 1,200 min) using the proc GLIMMIX procedure and a Tukey post hoc adjustment with cat as subject, time as the repeated measure. All data are presented as least square mean ± SEM with significance set as *P* < 0.05 and trends identified with a *P*-value between 0.05 and 0.09.

## Results

All cats tolerated the test diets with no adverse effects. Mean BW, daily food intakes, and daily macronutrient intake for each group are shown in Table 2. The BW was not affected by treatment and remained stable for all cats (*P* = 0.6082). Daily food intake was greater for cats consuming the LF diet compared to LP and LC (*P* < 0.0001), but energy intake as ME from the modified Atwater equation was not different between test diets (*P* = 0.1191). Energy intake as ME using the NRC predictive equations was significantly greater for the LP diet compared to the LF and LC test diets (*P* < 0.0001). Macronutrient intakes were different between test diets; protein intake was lower in LP compared to LC and LF (*P* < 0.0001), NFE intake was lower in LC compared to LF and LP (*P* < 0.0001), and fat intake was lower in LF compared to LC and LP (*P* < 0.0001) as intended.

**Table 2.** Mean weekly BW and daily intakes of food, energy, and macronutrients for cats (*n* = 12) consuming an LP, LF, or LC, extruded test diet for 2 wk^1^

Parameter	LP (*n* = 12)	LF (*n* = 12)	LC (*n* = 12)	*P*-value
Body weight, kg	4.97 ± 0.24	4.98 ± 0.24	4.97 ± 0.24	0.6082
Food intake, g/d	58.3 ± 1.0^a^	61.6 ± 1.0^b^	57.7 ± 1.0^a^	<0.0001
Energy intake, kcal/d, based on ME_Atwater_^2^	227.9 ± 2.0	223.8 ± 2.2	228.4 ± 2.0	0.1191
Energy intake, kcal/d, based on ME_NRC_^3^	269.1 ± 2.4^a^	241.9 ± 2.2^b^	242.9 ± 2.2^b^	<0.0001
Protein intake, g/d	18.2 ± 0.3^a^	26.0 ± 0.4^b^	23.0 ± 0.4^c^	<0.0001
NFE intake, g/d	23.1 ± 0.4^a^	20.1 ± 0.3^b^	16.9 ± 0.3^c^	<0.0001
Fat intake, g/d	10.6 ± 0.2^a^	7.6 ± 0.1^b^	10.7 ± 0.2^a^	<0.0001

^1^LC = low-carbohydrate, LF = low-fat, LP = low-protein, NFE = nitrogen-free extract, SEM = standard error of the mean.

^2^ME_Atwater_ calculated using modified Atwater Equation (Equation 2) (National Research Council, 2006).

^3^ME_NRC_ calculated using NRC Predictive Equations (Equation 3) (National Research Council, 2006).

^a^,

^b^,

^c^Superscript letters (a,b,c) denote significant differences between groups with different letters where a *P*-value < 0.05 is considered significant. No superscript letters in a row indicates no significant differences between groups for the measured parameter.

### Substrate utilization

Dietary treatment affected RQ values in the fasted, immediate postprandial, and postabsorptive state (Table 3). In the fasted state and as expected, RQ values were lower with LC diets compared to LF (*P* = 0.0154) and this was consistent when adjusted for BW (*P* = 0.0158). After a Tukey post hoc adjustment, a trend for a lower fasted RQ for cats consuming the LC diet compared to the LP diet was observed. Cats fed LC diets had a lower RQ in the immediate postprandial and postabsorptive state compared to LF diets (*P* = 0.0346 and *P* = 0.0407, respectively). This was also observed when adjusted for BW (*P* = 0.0354 and *P* = 0.419, respectively). No differences between the LP and LF diets in the fasted, immediate postprandial, or postabsorptive states were observed, and no differences between LP and LC diets in the immediate postprandial or postabsorptive states were noted (*P* > 0.05).

**Table 3.** Mean respiratory quotient for cats (*n* = 12) fed an LP, LF, or LC, extruded test diet for 2 wk^1^

Parameter	LP (*n* = 12)	LF (*n* = 12)	LC (*n* = 12)	SEM	*P*-value
RQ_Fasted_	0.78^a^^,^^b^	0.79^b^	0.76^a^	0.01	0.0154
* Adjusted for BW*	0.78^a^^,^^b^	0.79^b^	0.76^a^	0.01	0.0158
RQ_Immediate Postprandial_	0.73^a^^,^^b^	0.74^b^	0.72^a^	0.01	0.0346
* Adjusted for BW*	0.73^a^^,^^b^	0.74^b^	0.72^a^	0.01	0.0354
RQ_Post-absorptive_	0.81^a^^,^^b^	0.82^b^	0.79^a^	0.01	0.0407
* Adjusted for BW*	0.81^a^^,^^b^	0.82^b^	0.79^a^	0.01	0.0419

^1^BW = body weight, LC = low-carbohydrate, LP = low-protein, LF = low-fat, RQ = respiratory quotient, SEM = standard error of the mean.

^a^,

^b^Superscript letters (a,b) denote significant differences between groups with different letters where a *P*-value < 0.05 is considered significant. No superscript letters in a row indicates no significant differences between groups for the measured parameter.

**Figure 1.**
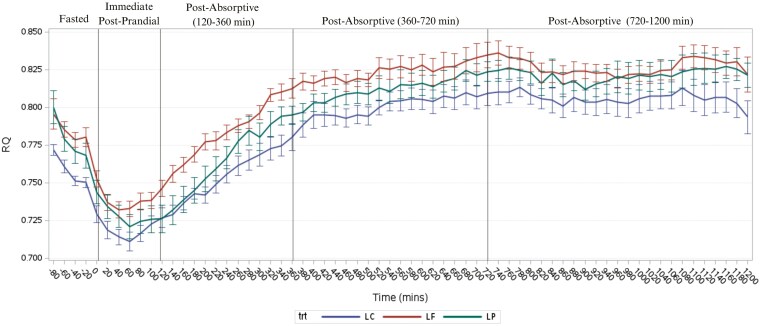
Mean RQ of cats consuming an LP (*n* = 12), an LF (*n* = 12), or an LC (*n* = 12), extruded test diet after 2 wk. Measurements of RQ were taken at 20 min intervals over a 20-h indirect calorimetry session. Food was provided at time 0. Vertical lines indicate the fasted (−60 to 0 min), immediate postprandial (0 to 120 min), and the postabsorptive periods (120 to 360, 360 to 720, 720 to 1,200 min).

Over time, the fasted RQ values for cats consuming the LP and LF diets were greater than the RQ in the immediate postprandial state (*P* < 0.0001), however, after a Tukey post hoc adjustment, cats consuming a LC diet had a trend towards a lower immediate postprandial RQ compared to fasted RQ values (Fig. 1). For all 3 dietary treatments, mean RQ from 120 to 360 min postabsorptive increased from the immediate postprandial state (0 to 120 min) (*P* < 0.0001). The RQ for cats consuming the LP, LF, and the LC diets was also greater at 360 to 720 min compared to the fasted, immediate postprandial, and from 120 to 360 min postabsorptive states (*P* < 0.0001). However, the RQ for all 3 dietary treatments appeared to plateau from 360 to 720 min postabsorptive and 720 to 1,200 min postabsorptive (*P* = 0.9012) and remained greater than in the fasted, immediate postprandial, and 120 to 360 min postabsorptive states (*P* < 0.0001). There were no differences detected for mean AUC values of RQ between LP, LF, or LC diets in the fasted state (*P* = 0.5689) (Table 4). A trend towards a greater AUC, from 0 to 120 min immediate postprandial, was observed for LF compared to LC diets (*P* = 0.0713). Additionally, no differences were found between dietary treatments for AUC in the postabsorptive state; 120 to 360, 360 to 720, and 720 to 1,200 min between dietary treatments (*P* = 0.6418, *P* = 0.2852, and *P* = 0.4118, respectively).

**Table 4.** Mean area under the curve for respiratory quotient of cats consuming an LP, LF, or LC extruded test diet for 2 wk^1^

(RQ*min)	LP (*n* = 12)	LF (*n* = 12)	LC (*n* = 12)	*P*-value
AUC_RQ_ (fasted)	47.16 ± 0.86	47.19 ± 0.86	45.91 ± 0.84	0.5689
AUC_RQ_ (0 to 120 min, immediate post-prandial)	87.18 ± 0.88	89.14 ± 0.88	87.01 ± 0.88	0.0713
AUC_RQ_ (120 to 360 min, postabsorptive)	190.25 ± 4.82	194.04 ± 4.91	186.82 ± 4.73	0.6418
AUC_RQ_ (360 to 720 min, postabsorptive)	285.60 ± 4.44	291.09 ± 4.44	281.41 ± 4.44	0.2852
AUC_RQ_ (720 to 1,200 min, postabsorptive)	391.52 ± 5.12	392.88 ± 5.12	385.16 ± 5.12	0.4118

^1^AUC_RQ_, area under the curve for respiratory quotient; LC, low-carbohydrate; LF, low-fat; LP, low-protein; RQ, respiratory quotient.

### Energy expenditure

**Figure 2.**
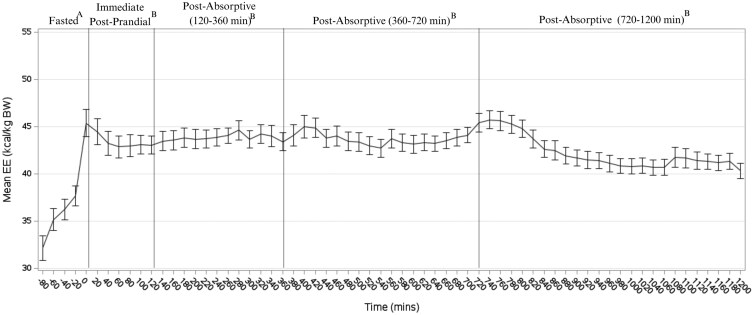
Pooled mean EE (±SEM) over time of cats (*n* = 12) consuming either an LP, LF, and LC extruded test diet for 2 wk. Vertical lines indicate the fasted (−60 to 0 min), immediate postprandial (0 to 120 min), and the postabsorptive periods (120 to 360, 360 to 720, 720 to 1,200 min). Different letters (A,B) denote significant differences (*P* < 0.05). BW, body weight.

Following feeding, EE was significantly greater for all cats compared to fasted EE as expected (*P* < 0.0001) (Fig. 2). The mean EE remained greater than fasted EE for the postabsorptive state (120 to 360, 360 to 720, and 720 to 1,200 min) (*P* < 0.0001), though no differences in mean EE were observed between the immediate postprandial and postabsorptive periods (*P* < 0.05). No effects of diet were observed on EE in the fasted (LP, 34.42 kcal/kg BW; LF, 33.17 kcal/kg BW; pooled SEM ± 1.86; LC, 35.62 kcal/kg BW) (*P* = 0.3977), immediate postprandial (LP, 42.73 kcal/kg BW; LF, 43.81 kcal/kg BW; LC, 44.08 kcal/kg BW; pooled SEM ± 2.04) (*P* = 0.880), or postabsorptive state (LP, 42.15 kcal/kg BW; LF, 43.81 kcal/kg BW; LC, 43.40 kcal/kg BW; pooled SEM ± 1.32) (*P* = 1370). There were also no effects observed when EE in the fasted, immediate postprandial, or postabsorptive states were adjusted for BW (*P* > 0.05). The RFMR for cats consuming the LP, LF, and the LC diet was 35.83 ± 1.10 kcal/kg BW, 35.83 ± 1.10 kcal/kg BW, and 37.17 ± 1.10 kcal/kg BW, respectively, with no differences observed between dietary treatments (*P* = 0.2747).

### Heat increment of feeding

The HIF amounted to 1.2%, 1.1%, and 1.1% of ME for the LP, LF, and LC diets, respectively, and did not differ between diets when using ME_Atwater_ or ME_NRC_ (*P* = 0.9106 and *P* = 0.9077, respectively) for the immediate postprandial period (Table 5). Similarly, the HIF over the entire post-feeding period (0 to 1,200 min) was not different between dietary treatments (*P* = 0.5481), amounting to 10.7%, 11.6%, and 11.2%, of ME_Atwater_ for the LP, LF, and LC diets, respectively. However, the HIF over the entire post-feeding period (0 to 1,200 min) amounted to 8.2%, 10.1%, and 9.7% of the ME_NRC_ for the LP, LF, and LC test diets, respectively, with the LP diet being significantly lower than the LF and LC diets (*P* = 0.0360). No differences were observed for the HIF per 100 g DM in the 2-h after feeding between dietary treatments (*P* = 0.5976) or over the entire post-feeding period (0 to 1,200 min) (*P* = 0.7632).

**Table 5.** Mean HIF of cats consuming an LP, LF, or LC extruded test diet for 2 wk^1^

(RQ*min)	LP (*n* = 12)	LF (*n* = 12)	LC (*n* = 12)	SEM	*P*-value
HIF_Immediate postprandial_					
HIF, % ME_Atwater_^2^	1.2	1.1	1.1	0.2	0.9106
HIF, % ME_NRC_^3^	0.8	0.8	0.7	0.2	0.9077
HIF, kcal/100g DM	5.1	4.3	4.8	0.9	0.5976
HIF_Entire post-feeding_					
HIF, % ME_Atwater_^2^	10.7	11.6	11.2	0.8	0.5481
HIF, % ME_NRC_^3^	8.2^a^	10.1^b^	9.7^a,b^	0.7	0.0360
HIF, kcal/100g DM	45.5	44.7	47.1	3.1	0.7632

^1^HIF = heat increment of feeding, LC = low-carbohydrate, LF = low-fat, LP = low-protein, RQ = respiratory quotient, SEM = standard error of the mean.

^2^ME_Atwater_ calculated using Equation 2 (National Research Council, 2006).

^3^ME_NRC_ calculated using Equation 3 (National Research Council, 2006).

## Discussion

This study utilized a 3 test diet approach following an isoenergetic reduction of macronutrients as previously described (Verbrugghe et al., 2010) to elucidate the individual effect of each macronutrient, providing insights into the short-term effects of LP, LF, or LC diets on RQ, EE, and HIF in adult cats and contribute to the growing knowledge of macronutrient metabolism and energy metabolism in cats.

Cats fed LC food had the lowest RQ values at all measured timepoints compared to LF. These findings were expected and follow similar previous reports of RQ responses to dietary macronutrients in cats (Lester et al., 1999; Hoenig et al., 2007; Gooding et al., 2014; Asaro et al., 2018), providing further support for cats’ ability to adapt to dietary macronutrient intake. Further, a reduction in RQ suggests greater reliance on fatty acid oxidation (Widmaier et al., 2016), and in humans a higher fasted RQ is associated with greater BW gain and body fat mass (Zurlo et al., 1990; Marra et al., 1998; Weyer et al., 2000; Ellis et al., 2010; Goldenshluger et al., 2021). Thus, a lower fasting RQ is thought to be beneficial in diverting fats from deposition as adipose tissue via lipogenesis and potentially contribute to obesity prevention in cats. In addition, the influence of dietary macronutrients on mitochondrial function and oxidative capacity has garnered interest. In rodents fed a high-fat diet, greater mitochondrial biogenesis and fatty acid oxidative capacity in skeletal muscle were observed (Turner et al., 2007; Hancock et al., 2008). Intracellularly, the use of fats requires oxygen whereas glucose does not, as such, if the cell has an aerobic limit, then it could reduce the ability of the cell to oxidize fat. The relationship between obesity and insulin resistance, as well as dietary macronutrients, to mitochondrial function, is unclear. Insulin resistance and type II diabetes in humans have up to a 30% reduction in skeletal muscle mitochondria and reduced aerobic capacity (Kelley et al., 2002; Petersen et al., 2004; Asmann et al., 2006; Gundersen et al., 2020). In the immediate postprandial state, RQ in obesity or type II diabetes appears conflicting, though studies have used a variety of dietary treatments (Galgani et al., 2008). Some reports suggested a greater RQ under these conditions and reduced metabolic flexibility in response to a meal (De Pergola et al., 2003; Lattuada et al., 2005; Galgani et al., 2008), and others observed no differences (Weinsier et al., 1995, 2002; Blaak et al., 2006; Siervo et al., 2016). It is not clear whether obesity and insulin resistance are, in part, a result of reduced mitochondrial function or vice versa, and further, how dietary macronutrients play a role (Asmann et al., 2006; Turner et al., 2007; Galgani et al., 2008; Hancock et al., 2008) It is thought that a dysfunction in the phosphorylation of AMP-activated protein kinase (AMPK) precedes insulin resistance in humans (Ruderman and Prentki, 2004). The AMPK pathway, as well as the mTOR pathway, are important regulators for mitochondrial biosynthesis and substrate utilization wherein activation of AMPK promotes fatty acid oxidation and stimulates glucose uptake in skeletal muscle. The understanding of role of these pathways in EE, substrate utilization, and dietary macronutrients in cats is limited and the findings of the present study cannot confirm whether similar changes occurred in the cats. The present study utilized indirect calorimetry methods as a non-invasive method to investigate energy metabolism in cats. However, future feline studies should consider metabolomics and gene expression to build upon these findings and enhance our understanding of the idiosyncrasies of feline metabolism.

In cats, increasing dietary fat intake, while maintaining energy balance, could aid in mitochondrial function and oxidative capacity similar to rodents (Turner et al., 2007; Hancock et al., 2008); however, studies investigating the role of dietary fat content, and the type of dietary fat, on mitochondrial biogenesis and gene regulation are lacking in cats. When fed to maintain BW, dietary fat could be beneficial in energetic efficiency, though excess supply of energy, often accomplished by free-feeding management practices with a high-fat commercial food in cats, can lead to increasing adipose tissue stores, obesity, and insulin resistance (Han et al., 1997; Nguyen et al., 2004a; Backus et al., 2007; Hancock et al., 2008). Although cats in the present study were not considered insulin resistant, the body condition varied from lean to overweight, therefore, insulin sensitivity may have been less pronounced in the overweight cats. Metabolic flexibility of cats was observed in the present study, though future studies should investigate how insulin resistance, obesity, and other metabolic diseases could affect mitochondrial function, oxidative capacity, and RQ. Further, the distribution of fatty acids was not assessed in the present study, though fatty acid type may affect the propensity for changes in mitochondrial efficiency (Turner et al., 2007; Hancock et al., 2008). Although the same ingredients were used in the present study, differing levels of ingredients may alter fatty acid ratios and, indeed, fatty acid concentrations, which could affect metabolic effects. Therefore, the type of fatty acids should be further investigated in future studies. Additionally, the obligate carnivore nature of cats needs to be considered. Indeed, the RQ of cats in the present and previous studies (Asaro et al., 2018; Godfrey et al., 2022; Rankovic et al., 2022) demonstrated different responses to a meal compared to omnivorous species such as humans, rodents, swine, and dogs. In cats, the RQ appears to decrease immediately following a meal, with carbohydrate oxidation increasing later, compared to humans, wherein the RQ increases immediately following a meal due to greater carbohydrate oxidation. Thus, comparisons between omnivorous species and cats should be made with caution.

Cats in the present study had EE values in line with previous literature (Gooding et al., 2014; Asaro et al., 2019) and the RFMR was also similar to previously published values (Nguyen et al., 2004b; Asaro et al., 2019). Nguyen et al. (2004b) had used the least observed metabolic rate (**LOMR**) in cats which was measured as 39.0 ± 1.0 kcal/kg BW per day whereas Asaro et al. (Asaro et al., 2019) used the RFMR which ranged from 35.4 kcal/kg BW d^−1^ to 36.5 kcal/kg BW d^−1^. The RFMR is the lowest value of EE observed and is intended to approximate the basal metabolic rate, similar to the LOMR which is the lowest amount of energy expended in the postabsorptive state at rest (Blaxter, 1989; Nguyen et al., 2004b). Measurements for the basal metabolic rate require an animal to restrain from physical activity and be in a postabsorptive state which was not possible due to ethical constraints in feline research, therefore, the RFMR was used. In the present study, the RFMR was not different among diets.

Similar to previous research in cats and other mammals, the EE immediately following a meal increased compared to the fasted EE in all cats (Asaro et al., 2019). Interestingly, cats from the Asaro et al. (2019) study exhibited a return to fasted EE values within 2 to 4 h postprandially with an increase in EE towards the end of the calorimetry period, whereas cats in the present study maintained an elevated EE throughout the postabsorptive period. An increase in EE towards the end of the calorimetry period could have been attributed to potential disturbances caused by the excitement of cats when researchers enter the room as observed in previous studies (Rand et al., 2002; Asaro et al., 2018). Postprandial EE curves similar to the present findings have previously been observed in this colony of young adult male cats (Camara et al., 2020; Rankovic et al., 2022) as well as in adult, middle-aged cats of mixed sex (Camara et al., 2020). Cats in previous studies were of ideal body condition; whereas body condition ranged from ideal to overweight according to BCS (Laflamme, 1997; Freeman et al., 2011) in the present study. Additionally, cats in the present study were colony cats wherein previous studies have included the provision of lipotropic supplements during growth and at maturity (i.e., choline and l-carnitine) (Godfrey et al., 2022; Rankovic et al., 2022, 2023). Although dietary treatments did not significantly alter EE in the previous studies, it is important to consider that long-term effects of lipotropic supplementation, as well as overall diet history, on oxidative capacity, energy efficiency, and EE have not been investigated in cats.

When fed to maintenance energy requirements, no differences were observed for EE in the fasted or postprandial state in cats consuming a high-fat versus high-carbohydrate diet (Gooding et al., 2014) and in the fasted state for cats consuming diets of differing carbohydrate levels (Asaro et al., 2018). Similarly, the present study did not observe EE to be affected by diet. This confirms that macronutrient intake does not directly influence EE, even when ingredients are controlled for. Rather, meal size and energy intake such as overfeeding or calorie restriction as well as feeding frequency can affect total daily EE (Apfelbaum et al., 1971; des Courtis et al., 2015; Camara et al., 2020). Indeed, a high-protein diet (protein, 47.3 %ME; fat, 44.5 %ME; NFE, 8.2 %ME) resulted in greater EE in overweight cats compared to a moderate protein diet (protein, 27.1 %ME; fat, 44.1 %ME; NFE, 28.8 %ME); however, in that study, the high-protein group had greater food and energy intake compared to the moderate protein group and thus, EE was likely influenced by meal size or energy content rather than dietary protein content (Wei et al., 2011). Additionally, long-term consumption (76 d) of a high-fat diet compared to a high-carbohydrate appeared to reduce postprandial EE in cats when fed to maintenance, though this coincided with an increase in BW and fat mass as well as a reduction in lean body mass upon which EE is largely controlled by (Center et al., 2011; Gooding et al., 2015). Together, the findings from Gooding et al. (Gooding et al., 2015) suggest that cats in that study shifted from lipid oxidation to lipogenesis over long-term exposure to a high-fat diet. However, while cats were fed equal amount of predicted ME, an increase in BW suggested that the predicted ME content underestimated the true ME (Gooding et al., 2015; Asaro et al., 2017), similar to Asaro et al. (2018).

Following current industry recommendations (AAFCO, 2021), the test diets in the present study were formulated and offered an allotment equal to their maintenance energy requirements based on the ME_Atwater_. Using ME_NRC_ resulted in greater ME values for each test diet, and alterations in the macronutrient distributions as a %ME. This is not surprising, as ME_Atwater_ tends to underestimate the ME of pet foods (Kienzle et al., 1998; Laflamme, 2001; Yamka et al., 2007; Hall et al., 2013; Asaro et al., 2017), though in certain instances, such as comparatively low macronutrient digestibility, it can overestimate the ME. Energy intake, when measured using ME_NRC_ was greater in cats consuming LP. Energy intake has previously been shown to affect EE. It is unclear why no difference was observed in the present study, though the short-term (2-wk) design could have been a factor, as could the variation in body condition among cats or alternatively, the digestibility of the diets. Further, test diets were formulated to have similar fiber and vitamin and mineral content; however, these were not analyzed upon manufacturing. Therefore, the concentrations of vitamins and minerals may have differed between diets. Recent reports suggest that greater antioxidant intakes in cats and dogs can reduce DNA damage and preserve mitochondrial function and cell production (Jewell et al., 2024) which could affect RQ and EE as discussed above. Indeed, in humans vitamin and mineral supplementation, such as vitamin E, D, and calcium, have been shown to increase EE (Li et al., 2010; Alaei-Shahmiri et al., 2020; Soares et al., 2022).

Although calorimetry is often used in dietary intervention studies for cats to assess RQ and EE, calculation of HIF is often lacking. The HIF represents the metabolic cost of consuming, digesting, and absorbing energy. In one study, the HIF of diets with varying perceived glycemic index levels was calculated from 0 to 2-h and 0 to 20-h postprandially (Asaro et al., 2019). Diets of high, moderate, and low perceived glycemic index had an HIF of 1.58 to 2.03 %ME (5.82 to 8.87 kcal/100 g DM) from 0 to 2-h postprandial, and from 19.5 to 21.7 %ME (77.6 to 94.6 kcal/100 g DM) from 0 to 20-h postprandial (Asaro et al., 2019). Although HIF in both time periods was lower in the present study, these values were on par with other carnivorous species (Smith et al., 1978; Chappell et al., 1997). Additionally, calorimetry results from previous studies in cats had calculated mean HIF (kcal/100 g DM) values of 3.93 ± 0.86 (Godfrey et al., 2022), 3.55 ± 0.50 (Rankovic et al., 2022), and 6.39 ± 0.86 (Rankovic et al., 2023). The HIF values from the present study are thereby in line with these values.

Dietary macronutrient density has previously been reported to affect thermogenesis in both humans and animals. In humans, dietary protein is reported to be the greatest contributor to HIF, followed by carbohydrate, and then fats. This was also observed in salmonoids, a carnivorous species (Smith et al., 1978; Westerterp, 2004). Indeed, in salmon, an increase in HIF was associated with intake of purified protein or carbohydrate bolus (Smith et al., 1978). Alternatively, an increase in dietary fats has previously been shown to reduce the HIF when measured over a 24-h calorimetry (Smith et al., 1978; Li et al., 2018). When using the ME_Atwater_, the 20-h HIF (%ME) followed a similar pattern numerically wherein the LF diet had the lowest fat content and greatest 24-h HIF, followed by the LC diet, which ha**d** greater protein than the LP diet. However, when using ME_NRC_, this pattern was significant wherein the LF food resulted in the greatest 20-h HIF (%ME), followed by LC and LP, respectively. No significance for the 2-h HIF (%ME) for either ME equation was likely due to the larger utilization of dietary fats in cats in the immediate postprandial period, as shown with the RQ curves in the present and previous studies (Gooding et al., 2014; Asaro et al., 2019). Thus, due to the metabolic patterns of cats wherein macronutrient utilization changes throughout the 20-h post-meal period, this suggests that 20-h HIF values should be utilized in future studies for cats. Additionally, use of ME_NRC_ provided a more accurate method of determining the ME and, therefore, changes in HIF (%ME).

It is important to note that cats in the present study exhibited a large variation in body condition, ranging from ideal to overweight. It is unclear how body condition impacts the HIF; however, gastric emptying and digestibility—all of which have been shown to differ between obese and non-obese humans (Wisén and Johansson, 1992; Davis et al., 2020; Steenackers et al., 2021)—can affect the HIF (Blaxter, 1989; McCue, 2006). A post hoc power analysis for 20-h HIF indicated a low statistical power (Power = 0.10). Controlling for body condition could improve the statistical power, though future studies should investigate body condition and HIF in cats. Additional factors such as meal frequency, age, as well as changes in temperature and humidity should be considered for future investigations of HIF, EE, and RQ.

In summary, the present study examined the short-term effects of an LP, an LF, and an LC diet in adult male cats. The findings indicated that RQ responds to short-term feeding of diets low in protein, fat, and carbohydrates in cats, with LC diets resulting in lower RQ values. Consistent with previous literature, dietary macronutrient intake did not directly impact EE when fed to maintain BW. Additionally, this study contributes to the limited research on HIF in cats demonstrating a lower HIF in cats similar to other carnivorous species. The present study further contributes to the large body of evidence that the ME_Atwater_ is inappropriate, and that the ME_NRC_, a more accurate measure, provides different results in terms of energy intake and HIF. The results could contribute to future research in cats to deepen the understanding of the role of macronutrients in feline metabolism and health.

## Abbreviations

AAFCO: Association of American Feed Control Officials
AMPK: AMP-activated protein kinase
AOAC: Association of Official Analytical Chemists
AOCS: American Oil Chemist Society
AUC: area under the curve
BCS: body condition score
BW: body weight
DE: digestible energy
DM: dry matter
EE: energy expenditure
GE: gross energy
HIF: heat increment of feeding
LC: low-carbohydrate
LF: low-fat
LP: low-protein
LOMR: least observed metabolic rate
LSM: least square means
ME: metabolizable energy
NFE: nitrogen-free extract
NRC: National Research Council
RFMR: resting fed metabolic rate
RQ: respiratory quotient

## Notes

*Conflict of interest statement.* The authors declare no conflicts of interest. H.G. declares that they have participated in paid internships and engagements with pet food companies within Canada. A.K.S. is the Champion Petfoods Chair in Canine and Feline Nutrition, Physiology, and Metabolism, consults for Champion Petfood, was previously employed by P&G and Mars Pet Care, serves on the Scientific Advisory Board for Trouw Nutrition, and has received honoraria and research funding from various commodity groups, pet food manufacturers, and ingredient suppliers. A.V. is the Royal Canin Veterinary Diets Endowed Chair in Canine and Feline Clinical Nutrition and declares that they serve on the Health and Nutrition Advisory Board for Vetdiet. A.V. has also received honoraria and research funding from various pet food manufacturers and ingredient suppliers. D.K. and J.S.B. declare that they are employed by Champion Petfoods Holdings and J.K. was employed by Champion Petfoods at the time of study trial and manuscript writing. At the time of publication, J.K. was employed by Mars Pet Nutrition. The authors declare that these do not conflict with respect to the research, authorship, and/or publication of this article.
